# Impact of Healthcare-Associated Infections Connected to Medical Devices—An Update

**DOI:** 10.3390/microorganisms9112332

**Published:** 2021-11-11

**Authors:** Nitin Chandra Teja Dadi, Barbora Radochová, Jarmila Vargová, Helena Bujdáková

**Affiliations:** Department of Microbiology and Virology, Faculty of Natural Sciences, Comenius University in Bratislava, 84215 Bratislava, Slovakia; d1@uniba.sk (N.C.T.D.); vargova301@uniba.sk (J.V.)

**Keywords:** nosocomial infection, medical devices, catheter, ventilation, microorganisms, biofilm, treatment

## Abstract

Healthcare-associated infections (HAIs) are caused by nosocomial pathogens. HAIs have an immense impact not only on developing countries but also on highly developed parts of world. They are predominantly device-associated infections that are caused by the planktonic form of microorganisms as well as those organized in biofilms. This review elucidates the impact of HAIs, focusing on device-associated infections such as central line-associated bloodstream infection including catheter infection, catheter-associated urinary tract infection, ventilator-associated pneumonia, and surgical site infections. The most relevant microorganisms are mentioned in terms of their frequency of infection on medical devices. Standard care bundles, conventional therapy, and novel approaches against device-associated infections are briefly mentioned as well. This review concisely summarizes relevant and up-to-date information on HAIs and HAI-associated microorganisms and also provides a description of several useful approaches for tackling HAIs.

## 1. Introduction

Healthcare-associated infections (HAIs) are nosocomial-acquired infections (hospital-acquired infections) that are not present in the patient before their hospitalization [1,2]. HAIs can occur in various wards during treatment; they are most often associated with hospitalization in intensive care units (ICUs). In ICUs, patients have a 5 to 10 times higher risk of acquiring an HAI due to both intrinsic (immunodeficiency) and extrinsic factors (the administration of medical devices). In addition, an ICU is often an epicenter of microorganisms with multi-drug resistance (MDR) [3].

HAIs typically occur from 48 h to 30 days post-treatment [4,5]. In Europe, the European Centre for Disease Prevention and Control (ECDC) created in 2005 “The Healthcare-Associated Infections Surveillance Network”, and in the USA, the World Health Organization (WHO)’s section on prevention of hospital-acquired infections published their practical guide (second edition) in 2012. The WHO have stated that HAIs are a global phenomenon responsible for a high amount of morbidity and mortality all over the world [6]; they are a major problem not only in developing countries but also in the highly developed countries in Europe [4,7,8]. The ECDC reported that around 3.2 million patients acquired HAIs in European Union (EU) countries, and among them, 37,000 mortalities have been registered every year [9]. These infections also cause economic losses in the hospital sector, which have been increasing every year. A survey performed by Duszynska et al. (2020) revealed that one in five patients are diagnosed with a device-associated HAI, which leads to an additional financial burden of over 10,000 euros per patient [10]. Hopmans et al. (2020) conducted a biannual point-prevalence surveillance on HAIs from 2007 to 2016 and identified a decrease in the incidence of HAIs in hospitals in the Netherlands, specifically in surgical site infections (SSIs) and urinary tract infections (UTIs). This survey showed a reduction in the mean length of hospital stay by 3 days, but the usage of antibiotics increased from 31% to 36%. In this way, they demonstrated the importance of surveys to know the impact of ongoing treatment procedures to help and modify those procedures to provide a high standard of healthcare [11].

**Figure 1:**
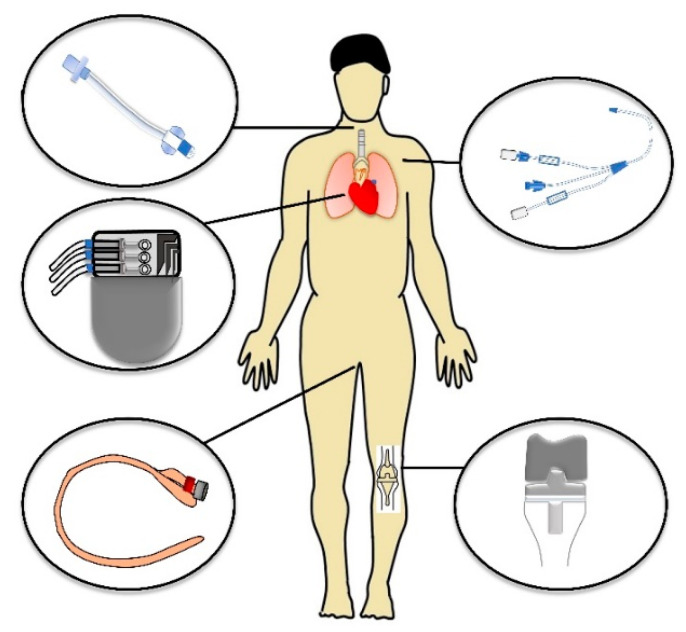
Schematic diagram of the most common locations of medical devices.

HAI is most often a result of using invasive procedures (i) such as the administration of temporary indwelling devices, for example, central venous catheters (CVCs), urinary catheters, vascular access devices, endotracheal tubes, tracheostomies, enteral feeding tubes, and wound drains, etc., or could emerge as a complication after surgical intervention associated with the administration of implants (ii). These SSIs can occur after an operation if no implant is left in place because of infected equipment or after the implantation of specific permanent devices, e.g., cardiovascular or orthopedic ones. Figure 1 summarizes the most common locations, where medical devices are administered.

HAIs comprise a wide range of infections categorized based on infected medical equipment. This includes central line-associated bloodstream infections (CLABSIs) and central venous catheter bloodstream infections (CVCBSI), catheter-associated urinary tract infections (CAUTI), and ventilator-associated pneumonia (VAP). The second group covers the above-mentioned SSIs [12].

Several studies have identified that the kind of microorganisms causing HAIs depends on the type of medical intervention and the medical device used as well as where it is administered in the human body [13,14].

Weiner-Lastinger et al. (2020) published a summary of data reported to the National Healthcare Safety Network from 2015–2017. They documented in total 311,897 HAIs and 356,633 associated pathogens. SSIs contributed the highest proportion of pathogens (43%), followed by CAUTIs (29%), CLABSIs (25%), and VAPs (3%). *Escherichia coli* proved to be the most common pathogen in HAIs, amounting to 18% of the identified pathogens. *Staphylococcus aureus* (12%) and *Klebsiella* spp. (9%) were observed as the second and third most detected pathogens, respectively [15].

The above-mentioned HAIs are frequently associated with the colonization of medical devices with microorganisms organized in biofilms [16]. The most predominant microbes are *S. aureus*, *Staphylococcus epidermis*, *Enterococcus* spp. from Gram-positive bacteria and *E. coli*, *Klebsiella pneumoniae*, and *Pseudomonas aeruginosa* from Gram-negative bacteria [12]. From the group of microscopic fungi, *Candida* spp. are the major causative agent of HAIs [17]. Single-species biofilms are frequent, but polymicrobial biofilms are not a rare phenomenon, even though there is less information due to the limits of detection techniques. Specific interactions between microorganisms enable different alterations of the morphology and/or physiology of cells. Small-colony variants or different non-cultivable states (e.g., viable but non-cultivable) can be induced after competitive interactions between some bacteria (e.g., *S. aureus* and *P. aeruginosa*) [18]. In addition, there is evidence for an increased number of persisters as well as the production of extracellular polymer substance (EPS) in mixed biofilms. However, establishing a direct link between the microorganisms organized in a biofilm, the colonization of medical devices, and infection in patients has still been problematic because of the lack of a guide, which clearly defines a biofilm as the source of the infection. Stewart and Bjarnsholt screened titles of articles in the Web of Science database for the terms “biofilm” together with “risk factor”; “biofilm” was mentioned 27,150 times and “risk factor” 37,617 times, but both terms were mentioned together only three times. The authors noted a critical comment that physicians who work directly with patients are detached from scientists focused on biofilm research conducted on medical implants [19].

The enhanced resistance of a biofilm to antimicrobial agents is another factor that makes a biofilm more virulent and often induces recurrent infections [12].

The immense impact of HAIs on the quality of healthcare provided as well as economic costs underlines the importance of this topic. The main motivation for writing this review was to provide a concise summary of the latest information on HAIs associated with medical devices, including the most frequently participating microbial pathogens and risk reduction possibilities and options.

## 2. HAIs Associated with Administration of Temporary Indwelling Devices

Several studies have identified that the kind of microorganisms causing an HAI depends on the type of medical implant inserted into the patient [17,20]. Additionally, it is necessary to determine precisely whether the patient got the infection before admission to the hospital or became infected during hospitalization. HAIs only include infections that do not appear in patients until 48 h after hospitalization. Evidence that the patient is infected are symptoms such as fever, chills, lethargy, cough, difficulty breathing, abdominal pain, and stool problems. Sepsis and inflammation may also be included among the common symptoms [21,22].

CVCs are considered to be a major source of bloodstream infections (BSIs) in hospitals, especially in patients with permanently impaired immunity. Their insertion is an invasive method and is used in both adult and pediatric patients [23]. Additionally, long-term intestinal problems in children also require the insertion of a CVC to provide parenteral nutrition, but this is frequently accompanied by infections resulting in an increased rate of morbidity and mortality [24].

Another type of common nosocomial infection caused by catheters in hospitals is CAUTI. Removal of the urinary catheter in the shortest time possible is a basic step to prevent infections. However, adhering to this rule in hospitals is very difficult. Long-term catheterization should only be recommended in very justified cases. From this perspective, identifying the risk factors for CAUTI is critical [25,26]. Letica-Kriegel et al. (2019), similarly to other authors, observed that when the duration of catheterization increased, the risk of infection also increased. Furthermore, they confirmed that approximately 12% of patients with an indwelling catheter developed CAUTI within 30 days. Some risk factors are predominantly associated with CAUTI [27,28]. The above-mentioned study defined two main hazards, i.e., sex (female) and those associated with mobility issues [27]. Medina-Polo et al. (2021) determined in their study that a catheter in the upper urinary tract and immunosuppression are the other critical factors contributing to CAUTI caused by MDR bacteria [29].

VAPs as part of intensive care are also particularly dangerous. Li et al. (2018) documented that bacterial contamination was largely observed on devices that were used repeatedly. Therefore, emphasis should be placed on the constant sterilization of intubation equipment used [30]. Patient monitoring can help physicians to detect changes in lung function in a timely manner to reduce the risk of VAP and mortality [31].

The predominant microorganisms isolated from the above-mentioned medical devices are Gram-positive bacteria of *S. aureus* and *S. epidermidis*, then enterococci and streptococci. The Gram-negative bacteria include *E. coli*, *Enterobacter* spp., *Acinetobacter baumanii*, *P. aeruginosa*, *K. pneumoniae*, and *Proteus mirabilis*, as well as representatives of the yeast from the genus *Candida* [12,17,20].

Microorganisms can survive not only in planktonic form, but also they frequently colonize medical devices and form mono- or inter-species communities. Biofilms have subsequently become more resistant to conventional drugs, often resulting in chronic infections in patients [17,32,33,34]. Svensson et al. (2021) noted that a strong biofilm production was significantly associated with recurrent infection [34]. According to a meta-analysis of PubMed and Web of Sciences databases from January 2005 to May 2020 published by Pinto et al. (2021), strong biofilm producers tested in vitro and associated with BSI were strongly represented among the resistant strains. Methicillin-resistant *S. aureus* (MRSA) was mainly mentioned. Moreover, biofilm producers were also highly linked to BSI persistence. It is of interest that this association was the highest for *Candida* spp. As for UTIs, multi-resistant *E. coli* was observed to be the predominant strong biofilm producer. The above-mentioned study clearly proved that biofilms must be assumed to be a BSI and UTI resistance factor [33].

### 2.1. Central Line-Associated Bloodstream Infections Including Catheter-Related Infections

The term “central line” is defined as an intravascular access device or catheter that terminates at or close to the heart or in one of the major blood vessels. CVCBSI is a clinical definition used when diagnosing and treating patients that identifies the catheter as the source of BSIs. Catheter colonization is defined as the presence of ≥15 Colony-Forming Units (CFU) of a single organism per catheter. The colonization of a catheter by microorganisms together with CLABSI can be considered to be catheter-related infections (CRI) [32,35].

The ECDC published an updated document providing definitions of standardized protocols of data collection and reporting for hospitals participating in the surveillance of HAIs in ICUs across Europe. A BSI should be reported as a CRI when the same microorganism is cultured from the catheter, or symptoms improve within 48 h after removal of the catheter, or a BSI is reported as secondary to another infection [3,14]. This definition differs from the definition of the National Healthcare Safety Network of the USA Centers for Disease Control and Prevention of CLABSI, which includes all primary BSIs with a central line in place for at least 48 h on the date of the BSI [36].

CVCs are generally inserted in around 50% of patients in ICUs. Because of direct contact with the bloodstream, there is a higher risk of septicemia leading to severe systemic infections, including sepsis. Therefore, the application of a CVC to a patient is the most frequent cause of hospital bacteremia, and about 40% of all primary BSIs are related to a CVC [13,35]. CVCs are tubular structures generally implanted into veins and used to deliver medication or nutrients to the patients, for hemodialysis and blood testing [37]. This CVC is inserted through a peripheral vein or a proximal central vein, internal jugular, subclavian, or femoral vein. There are four types of CVC: non-tunneled pulmonary artery CVC, peripherally inserted CVC, tunneled CVC, and totally implantable CVC [35].

Several European prevalence studies have been conducted by the ECDC [5]. Plachouras et al. (2018) summarized the data from the last study, conducted in 2015–2016, in which 11 European countries were involved. The data showed that from a total of 141,955 patients from 617 ICUs, 2555 CLABSIs were identified, corresponding to a cumulative incidence of 1.8 episodes per 100 patients and representing 48% of all ICU-acquired BSIs [38]. For comparison, the previous study, conducted by the European network for the surveillance of ICU-acquired infections in 2004—2005, collected data from nine countries. The incidence density of BSI ranged from 1 to 3.1 per 1000 patient days, where 60% of cases were diagnosed as CVCBSI [13]. Generally, rates of catheter-related bloodstream infections (CRBSIs) higher than 2 per 1000 catheter-days should not be acceptable [5,13]. The CRBSI-related mortality and the annual costs associated with CRBSI in the European countries according to the study were about 1000–1584 deaths per year and costs of 35.9 to 163.9 million euros [39]. The surveillance and introduction of bundles of care processes relating to the insertion and maintenance of CVCs produced a fall in the annual CRBSI from 3.4 to 0/1000 patient days with zero episodes [39]. Novosad et al. (2020) compared data collected from the USA on the incidence of CLABSI infections during the period of 2011 to 2017 and revealed that around 50% of all ICU patients were with CVCs. Of the CLABSI, around 60% and 40% were adults and children, respectively [40]. A case study conducted by Baier et al. (2020) showed that the healthcare costs of CLABSI analyzed in stem cell transplantation and cancer patients was estimated to amount to be 8810 € per case [41]. According to the CLABSI prevention guidelines developed by the Asia Pacific Society of Infection Control, a pooled incidence density of CLABSI reached a value of 4 to 7 per 1000 catheter-days [32].

Several factors, such as patient condition, duration of catheterization, type of catheter material, and catheterization handling have been shown to increase the risk of CVC infection [42]. A highly contributing factor is the duration of catheterization, while a significant proportion of CRIs occurred after ≥15 days [43]. As is mentioned in the “Guidelines for the Prevention of Intravascular Catheter-Related Infections”, the type of catheter material as well as insertion site has an impact on CLABSI occurrence. Catheter irregularities, the formation of a fibrin sheath, and thrombogenic potential could allow pathogenic microorganisms to adhere, resulting in biofilm formation [35]. For example, the use of polyvinyl chloride or polyethylene materials can cause less complications associated with infections compared to other materials (Teflon, polyurethane) or steel needles [44]. A comparative study by Pitiriga et al. (2020) confirmed this fact and showed that catheter insertion into the femoral vein was associated with a higher rate of BSI and catheter colonization compared to other sites such as a subclavian and internal jugular site. Another factor may be seasonal prevalence changes among microorganisms [43]. A study by Blot et al. (2021) examined a seasonal variation of monthly hospital-acquired BSIs, including CLABSIs incidence rates, in the period of 2000 to 2014, with summer incidence spikes for *Enterobacterales* (*E. coli*, *K. pneumoniae,* and *Enterobacter cloacae*) and non-fermenters (*P. aeruginosa*, *A. baumannii*, and *Stenotrophomonas* spp.) [45].

Microorganisms isolated from CVCs (also CLABSI) represent a range from virulent microorganisms to the normal resident microbiome of the skin at the insertion site. From Gram-positive bacteria, specifically coagulase-negative staphylococci (CoNS), namely *S. epidermidis,* then MRSA, enterococci (*Enterococcus faecalis* and *Streptococcus* spp.) were confirmed. From Gram-negative bacteria, *P. aeruginosa*, *K. pneumoniae*, *E. coli*, *Enterobacter* spp., *A. baumannii*, and *P. mirabilis* are frequent sources of CRIs. *Corynebacterium* spp. is less frequent. From yeasts, *Candida albicans* and other *Candida* spp. have been associated with catheter infections [13,46,47,48]. It is noteworthy that a high incidence of MDR related to CLABSI was observed [49]. In general, there are increasing trends of CVC infections caused by Gram-negative bacteria [50]. According to the Plachouras et al. (2018), the most commonly isolated microorganisms in ICU-acquired CLABSIs were Gram-positive bacteria, with 48.1%, and then Gram-negative bacteria with 42.6%. Other microorganisms were estimated to amount to 9.3%. The incidence among Gram-positive bacteria was as follows: CoNS (28.4%), *Enterococcus* spp. (9.3%), and *S. aureus* (8.7%). Among Gram-negative bacteria, *Klebsiella* spp. (11.4%) was the most common, followed by *P. aeruginosa* (7.4%). *Candida* spp. belonged to the group “Others”, with 8.8%. [51].

*C. albicans* is the most common *Candida* spp. with CVC-related infections. The mortality rate of these infections can reach 50%, even with treatment [52]. One of the most important virulence factors of *C. albicans* is the ability to switch from the yeast form to the hyphal form. This morphological switching is an advantage that provides an appropriate base for the colonization of hyphae by other microorganisms, especially *S. aureus*, *S. epidermidis*, or enterococci [53].

The above-mentioned microorganisms usually have a high capability to form biofilms. They can attach to both abiotic and biotic surfaces and transform from planktonic to sessile form and then aggregate into microcolonies embedded in EPS. In particular, microorganisms commonly isolated from BSIs, namely *Staphylococcus* spp. and *Candida* spp., are able to survive up to 20% in the polymicrobial community. In such mixed biofilms, *C. albicans* can contribute to increased bacterial resistance to antibiotics, the survival and growth of anaerobic bacteria under aerobic conditions or lead to an increase in their virulence. Moreover, these biofilms are much more resistant to treatment and the host immune response. Biofilms are therefore often responsible for the dangerous contraindications associated with CRI and can develop into a chronic condition [54].

Many techniques have been used to prevent the formation of biofilm by targeting different stages of biofilm formation. Conventional approaches for the prevention of CLABSI include general precautions such as aseptic maintenance during CVC insertion [55]. Conducting staff training and implementing strict guidelines in a few hospitals in the USA produced a significant reduction in CLABSI cases [23,56]. In case of infection, non-essential catheter removal should be considered [13,57]. Another well-known strategy, antibiotic lock therapy, is the most tested. A controlled release of the antibiotics assists the penetration of antimicrobial compounds through the barrier of a biofilm [58]. Other approaches for preventing CVC-related infection, such as antimicrobial CVC, needleless IV access devices, antimicrobial dressing, etc., are in use as well [59,60]. One of the antibiotic combinations used in the impregnation of CVC is minocycline/rifampin, or for infants, miconazole and rifampin [61]. The use of different coatings, such as chlorhexidine and gluconate or silver sulfadiazine, reduced the risk for CRBSI compared to the standard non-coated catheters [62]. However, the study of Cui et al. (2020) suggests that there is a doubt of whether sufficient antibacterial function can be maintained with prolonged duration of catheter placement due to the blood flow [63].

Another promising strategy is coatings comprising naturally occurring antimicrobial peptides with a strong antibacterial/antibiofilm potential [64]. Catheter lock therapy is useful, especially in cases of uncomplicated long-term catheter-related BSI caused by CoNS or *Enterobacteriaceae*. A catheter lock solution, taurolidine, has provided promising results among children as taurolidine prevents biofilm formation and has broad-spectrum bactericidal and antifungal activity [47]. Kumar et al. (2021) introduced this antimicrobial lock therapy as the best economically viable option. They utilized S-nitroso-N-acetyl-l-cysteine ethyl ester (SNACET) as the catheter lock solution as it generates nitric oxide, which manifests antimicrobial properties. Parameters such as stability and release were analyzed, and the efficacy of microbial adhesion against *S. aureus* and *E. coli* showed an over 99% reduction. SNACET has also been used for catheter coating. This catheter provided a 90% reduction in bacterial adhesion, providing a novel idea for tackling CVC infections [65]. Polysaccharides are interesting molecules for antifouling applications. Research conducted by Bujold et al. (2020) reported that low-dose unfractioned heparin decreased the incidence of CLABSI among critically ill children [66].

Experience obtained in material chemistry research suggests that a combination of antifouling and fouling release properties of a material could significantly improve its properties because of resistance to protein absorption and bacterial adhesion [67].

### 2.2. Catheter-Associated Urinary Tract Infection

UTIs are very common. Most UTIs are considered to be uncomplicated, well-treatable infections occurring in healthy individuals. Unlike them, complicated UTIs are associated with different serious diagnosis or when the infection is caused by a resistant microorganism that significantly increases the risk of therapy failure. UTIs are prevalent around the world and represent approximately 40% of hospital-acquired infections. They usually occur after instrumentation such as nephrostomy tubes, ureteric stents, suprapubic tubes, or Foley catheters. Among UTIs, 75% to 80% of infections are associated with using catheters [68,69]. The study of Gunardi et al. (2021) confirmed this observation: out of 109 catheterized patients, 78% with a urinary catheter were documented as being positive compared to only 37.62% of samples isolated from urine [70].

Urinary catheters are tubular structures made of latex or silicone material used to prevent retention during the patient’s surgical procedure. An indwelling catheter has a high risk of forming a biofilm compared to intermittent catheters present in the body for a short time (for up to 7 days) [23]. CAUTI is defined as a symptomatic UTI in a person with a catheter. This definition remains controversial, but is more or less globally accepted [69,71]. The prevention of CAUTI is discussed in the Centers for Disease Control and Prevention (CDC)/Healthcare Infection Control Practices Advisory Committee’s document “Guideline for Prevention of Catheter-associated Urinary Tract Infection”, which was updated in June 2019. The main mission of this guideline is to formulate recommendations and explicit links between the evidence and recommendations. The document includes key questions focused on (i) who should receive urinary catheters; (ii) benefits and risks; (iii) the best practices for preventing CAUTI [71]. The distinction between CAUTI and non-catheter-associated UTI is elaborated in the CDC/National Healthcare Safety Network document [72].

CAUTIs are responsible for causing mild catheter encrustation and bladder stones as well as septicemia, endotoxic shock, and pyelonephritis. Usually, a person with a catheter for over 30 days has been colonized, often with two to three types of microorganisms. As the main pathogens associated with UTIs are significant candidates for antibiotic treatment, they are a frequent source of horizontally transmitted resistance [73]. Management of these infections is important in two respects: (i) providing proper treatment, and (ii) reducing costs associated with an HAI [74]. Despite advances in prevention guidelines, there remains a lack of knowledge concerning the risk factors for CAUTI [75].

Gunardi et al. (2021) analyzed a bacterial suspension of 109 catheters and the urine of catheterized patients. They confirmed that gender and the duration of catheterization are the two main risk factors associated with CAUTI. The most frequently isolated microorganisms from a urinary catheter were *E. coli* (28.1%) followed by *Candida* spp. (17.8%), *K. pneumoniae* (15.9%), and *E. faecalis* (13.1%), and biofilm-producing microorganisms were observed in 40% of isolates. Surprisingly, they did not find a correlation between biofilm and age or diabetes mellitus, which have generally been known as risk factors of CAUTI. The authors also observed that bacteriuria prior to catheterization as a single parameter cannot be directly associated with the formation of a biofilm [70].

Microorganisms frequently colonize the surface of a urinary catheter, resulting in the formation of biofilms. Microorganism, such as *E. coli*, *Enterococci* spp., *S. aureus*, *P. aeruginosa*, *P. mirabilis*, and *Candida* spp. are also frequently isolated from the infected patient’s catheters [76].

Medina-Polo et al. (2021) determined that a catheter in the upper urinary tract and immunosuppression are the other critical factors contributing to CAUTI caused by MDR bacteria. They described that resistant bacteria occurred in 100 out of 438 (22.8%) positive cultures among patients with HAIs. The majority of resistant microorganisms were found in patients with a catheter in the upper urinary tract. Of the microorganisms, 28.4% of cultures were identified within the *Enterobacteriaceae* family (23.8% and 44.7% in *E. coli* and *Klebsiella* spp., respectively). As for resistance, 7% of *Enterobacteriaceae* showed resistance to carbapenems (1.3% and 10% for *E. coli* and *Klebsiella* spp., respectively). The rate of *P. aeruginosa* resistant to at least three antibiotic groups was 36.3% [29].

Several strategies have been introduced to tackle CAUTI. The most important is to reduce unnecessary catheterization and discontinue catheters as soon as possible. Patients with asymptomatic bacteriuria can generally be treated initially with catheter removal or catheter exchange and do not necessarily need antimicrobial therapy [77].

In infection, the treatment procedure should be based on the preliminary diagnosis and identification of biofilm-forming microorganisms, as biofilm manifests increased resistance compared to the free-floating planktonic cells. The minimal inhibitory concentration (MIC) of amdinocillin determined for *E coli* is 0.4 µg/mL, whereas 400 mg/kg is required for eradication of biofilm [78]. Experiences suggest that sub-lethal doses of antibiotics against *K. pneumoniae* biofilm can further promote resistance against an extended broad spectrum of beta-lactam antibiotics [79].

Vallée et al. (2017) utilized temocillin, which possesses an immense urinary and prostatic diffusion property. Moreover, it manifests a high bactericidal activity as it kills bacteria, producing an extended spectrum of beta-lactamases [80]. The research group of Ji et al. (2020) studied cranberry juice’s effectiveness on patients with UTI with short-term catheters and proved significant effects against the visible symptoms. Cranberry contains the active ingredient proanthocyanidins, which prevents the adhesion of microorganisms on the catheter surface. Cranberry was suggested as a supplemental therapy along with other strategies, which can assist in the reduction of the number of catheter days, providing a low chance of infection [81]. Costa et al. (2020) developed anti-adhesive coating catheters with a natural polymer produced by cyanobacteria. These coatings were further tested in culture media and artificial urine biofilm-promoting conditions against primary contaminants of UTIs. The results proved excellent anti-adhesive properties, resulting in a reduced attachment of microorganisms to the catheter surface. This could be a novel alternative strategy to reduce the usage of antibiotics [82].

The usage of nanoparticles in biomedical devices is a promising approach. Shalom et al. (2017) synthesized catheters coated with Zn-doped CuO nanoparticles. These catheters were tested in vivo in rabbits, and they provided excellent results in biofilm inhibition assays with no visible biofilm formation, even on the 7th day post-infection. Moreover, the biocompatibility tests proved the safety of the materials [83]. Another highly prominent alternative to the antibiotic strategy is the usage of antimicrobial peptides. Yu et al. (2017) developed a surface with active antimicrobial peptides labelled with cysteine on polyurethane. This surface coating prevented bacterial adhesion by up to 99.9% for Gram-positive and Gram-negative bacteria. The in vivo experiments conducted on a mouse model showed a 4-log reduction. These catheters also proved to be safe in cytotoxic studies with fibroblast cells [84]. Simple but effective techniques such as using chlorhexidine (0.1%) for cleaning prior to the catheter insertion can reduce the risk of infection. Switching to chlorhexidine from saline has saved AUD 387,909 per 100,000 for Australian hospitals [85]. The use of silver alloy in urinary catheter coatings to reduce CAUTI is being tried, but no clear significant benefit has been observed [86,87]. The usage of fosfomycin trometamol is possible for the treatment of CAUTI patients as it possesses a low rate of bacterial resistance, and no cross- or parallel-resistance with other frequently used antibiotics has been identified [88].

### 2.3. Ventilator-Associated Pneumoniae

VAP is the predominant infection of pulmonary parenchyma that develops in patients being supported by mechanical ventilators. Approximately one-third of nosocomial pneumonia cases, with the majority being VAPs, are acquired in the ICUs [89]. Patients are often in bad condition, and the invasive procedure of intubation may contribute to the patient’s risk of colonization by exogenous microbes. They can come from contaminated bronchoscopes, water supply, respiratory equipment, humidifiers, ventilator temperature sensors, respiratory nebulizers, or a contaminated environment [90]. The majority of infections occur within 48–72 h of subjecting the patient to a mechanical ventilator [91,92]. In previously healthy patients, normal oropharyngeal microbiota are usually involved in early-onset VAP (less than 4 days of hospitalization), whereas late-onset VAP (after at least 5 days of hospitalization) is more likely caused by nosocomial, often MDR pathogens [93].

VAPs cause high infection rates and have demonstrated a high morbidity and increase in healthcare costs, leading to a financial burden on patients. Epidemiological studies reviewed by Bassi et al. (2014) pointed to the development of VAPs in approximately 10 to 40% of the patients on mechanical ventilation for more than 2 days [90]. The estimated attributable mortality of VAPs is around 10%, with higher mortality rates in surgical ICU patients [94]. Incidence rates vary among countries, while discrepancies may be caused by different interpretations of definitions, the selected type of microbiological sampling method, or the studied population [92]. In addition, important differences between approaches in Europe and the USA have become apparent. International recommendations for hospital-acquired pneumonia (HAP)/VAP diagnosis, treatment and prevention (Guidelines for the management of HAP and VAP) were published under the auspices of a group of organizations (European Respiratory Society, European Society of Intensive Care Medicine, European Society of Clinical Microbiology, and Infectious Diseases and Asociación Latinoamericana del Tórax) [89]. In America, the CDC American Thoracic Society and Infectious Diseases Society of America established the clinical pulmonary infection score criteria, which are even used by many studies in Europe [95].

According to Torres et al. (2017), the incidence of VAPs is still very high (50%), despite the decreasing trend compared to the previous 10 years [96]. In EU countries (nine EU countries included, study conducted in 2006), an incidence density of 18.3 VAP episodes per 1000 ventilator days was noted [97]. The European study of ICU-acquired infections (data collected in 2007) showed that the overall incidence density was 7.9 pneumonia episodes per 1000 patient days. The mean device-adjusted pneumonia rate was 13.2 intubation-associated pneumonia per 1000 intubation days [98]. From recent national studies, Wałaszek et al. (2018) monitored VAPs in Polish ICUs from 2013 to 2015. The incidence of VAP was 8.0%, and the incidence density was 12.3/1000 ventilator days [99].

VAP risk factors include oropharyngeal and gastric colonization; thermal injuries; posttraumatic, postsurgical intervention factors such as emergency intubation, reintubation, tracheostomy, bronchoscopy, and inserting a nasogastric tube; patients’ body positioning; level of consciousness; stress ulcer prophylaxis; and the use of medications, including sedative agents, immunosuppression, and antibiotics [100]. There is still confusion in the epidemiology and diagnostic criteria of VAPs, despite major advances in microbiological tools and antimicrobial treatment regimens [92]. Modification of the oropharyngeal flora and trans-colonization plays a central role in the risk of infection. A better understanding of its physiopathology must be a priority in the context of the prevention of this infectious risk [101].

Microorganisms such as *P. aeruginosa*, members of the family *Enterobacteriaceae*, *A. baumannii*, *Stenotrophomonas maltophilia*, and MRSA have been regarded as culprits in these infections [102,103]. These microorganisms often form biofilm. In one of the above-mentioned studies, the predominant etiologic agents causing VAP were *Enterobacteriaceae* (32.6%), non-fermenting Gram-negative bacteria (27.6%), *S. aureus* (21.3%), and *K. pneumoniae* (12.5%). Using substandard methods, such as the cultivation of endotracheal aspirate or positive blood culture, *A. baumannii* (23.8%) was also commonly observed [99]. Similar microorganisms have been described in the work of Thorarinsdottir et al. (2020), who monitored the microbial composition (biofilm) colonizing mechanical ventilators [104].

According to a prospective observational study on intensive care nosocomial pneumonia in Europe, data on VAP incidence showed variability between the countries involved. In Spain, France, Belgium, and Ireland, the predominant isolate was *S. aureus*; *P. aeruginosa* was observed in Italy and Portugal; *Acinetobacter* was predominant in Greece and Turkey; and *E. coli* was the most frequently isolated in Germany [97]. Besides bacteria, *Candida* spp. are frequently found in bronchial samples in mechanically ventilated patients. The study of Timsit et al. (2019) showed that 68.5% of patients receiving mechanical ventilation had a tracheal colonization with *Candida* spp. Interaction between fungi and bacteria could lead to several complications (increase in bacterial toxin production or inflammation); however, the study did not find an association with a higher risk of bacterial VAP [105]. Another fungal pathogen such as *Aspergillus fumigatus* can be involved in VAP, particularly in patients with a recent history of influenza [106]. Recently, there have been studies showing that SARS-CoV-2 infection can also be associated with VAP. The incidence of ventilator-associated lower respiratory tract infections is significantly higher in patients with SARS-CoV-2 pneumonia compared to patients with influenza pneumonia or no viral infection upon ICU admission [107,108].

Minimal ventilator usage is considered a good strategy, and the treatment should be limited to 7 days in the vast majority of cases [92]. However, ventilator support is frequently necessary and critical for patients in the ICU. Several strategies have been in place against VAP. The administration of available antibiotics, for example, tobramycin inhalation solution, has reduced the recurrence of *P. aeruginosa* among ICU patients compared to the control group treated with systemic antibiotics in Japanese hospitals [109]. Sahuquillo et al. (2015) studied the effectivity of treatment by systemic antibiotics compared to their inhalation (tobramycin or colistin) in patients with microbiologically proved biofilms associated with VAP. Microbial infection was not influenced by the selected way of treatment [110].

Selective digestive decontamination (SDD) as well as oral hygiene care (OHC) seems to be important for reducing infections in critically ill patients with mechanical ventilation. Janssen et al. (2021) in their study showed that patients with SDD treated with a paste and suspension composed of amphotericin B, tobramycin, and colistin, administered orally, had a better prognosis in developing postoperative pneumonia, with 20.1% cases compared to 36.9% once without SDD [111]. Van Hout et al. (2019) treated patients four times a day with an oropharyngeal paste containing polymyxin E/colistin, tobramycin, and amphotericin B (2% concentration). Another group, in addition to the oropharyngeal paste, was treated four times per day with 10 mL of non-absorbable suspension of polymyxin E or colistin (100 mg), tobramycin (80 mg), and amphotericin B (500 mg) through a nasogastric tube. Additionally, a third-generation cephalosporin (cefotaxime, 1000 mg four times per day or ceftriaxone 2000 mg once per day) was administered intravenously during the first four days in ICU admission. The results showed that SDD significantly reduced mortality [112].

The link between VAP and dental plaque/biofilm has also been intensively studied. It was shown that brushing teeth with 0.12% chlorhexidine is also effective in inhibiting oral biofilms, reducing the incidence of VAP in patients [113]. Hua et al. (2016) tested chlorhexidine mouthwash on VAP-associated critically ill patients in the ICU and proved a reduction in VAP from 26% to approximately 18%. However, no evidence in mortality difference and duration of ventilation was obtained comparing patients with and without OHC [114].

The use of an endotracheal tube with an ultrathin and tapered cuff and coated with antimicrobial agents, such as hexetidine, silver, silver-sulfadiazine, and chlorhexidine, has also shown promising results [115]. An alternative strategy conducted by Prazak et al. (2019) showed the effectiveness of bacteriophages against resistant *S. aureus*. Animal experiments proved that curing infection was not absolute, but a significant reduction was observed [116]. A study by Homeyer et al. (2020) introduced the idea of functionalizing antibactericidal agents such as nitric oxide into the endotracheal tubes. This proved its efficacy by significantly reducing *P. aeruginosa* among ICU patients [117]. Using photodynamic inactivation to decrease biofilm formation on endotracheal tubes gave excellent results. Zangirolami et al. (2020) functionalized endotracheal tubes with the photosensitizer curcumin and then irradiated them with an external light source. This assay proved to be effective against both Gram-positive (*S. aureus*) and Gram-negative bacteria (*P. aeruginosa*, *E. coli*). Moreover, the application of this technique reduced biofilm thickness accompanied by a disruption of biofilm cells [118]. İbiş and Ercan (2020) used an interdisciplinary approach of using dielectric barrier discharge plasma and air plasma afterglow against biofilms formed by *P. aeruginosa*, *A. baumannii*, *S. aureus*, and *C. albicans*. In these protocols, the biofilms were significantly reduced without any significant damage to the endotracheal tube [119].

These studies show that various new alternative methods for the treatment of VAPs are constantly being developed. However, the priority is continuous patient monitoring to prevent chronic infections, and antibiotics should be given to the patient as soon as VAP is suspected.

## 3. HAI Associated with Surgical Site Infections

SSIs are infections that occur in the operation site of the patient. They can be superficial or more serious, involving tissues under the skin, organs, or implanted material [120]. If a device is implanted during surgery, the patient is at a higher risk of infections associated with a device. SSIs are among the most common HAIs. Moreover, SSIs are associated with an increase in the risk of mortality. The SSI does not significantly impact the degree of infection according to the pre-operation length of stay, but the post-operation length of stay is significant, providing data that reducing the number of days in hospital after surgery could reduce the occurrence of SSIs [121].

In the EU, the ECDC provides documents containing important definitions, protocols for monitoring, epidemiological reports, and guidance for the prevention and control of SSIs. The last epidemiological report published in 2019 collected data from 2017 of 12 EU members and one European Economic Area [122].

Nine types of surgical procedures are included: coronary artery bypass graft, open and laparoscopic cholecystectomy, open and laparoscopic colon surgery, caesarean section, hip prosthesis, knee prosthesis, and laminectomy. The percentage of SSIs varied from 0.5% to 10.1%, and the incidence density of in-hospital SSIs per 1000 post-operative patient days varied from 0.1 to 5.7, depending on the type of surgical procedure [122]. These infections cause tremendous financial losses as they account for 7 million pounds of losses in a hospital in England [123]. As these SSIs increase the duration of stays, additional costs for coronary artery bypass surgery patients (costing approximately 10,000 euros/patient in Germany) were caused by the prolonged usage of antibiotics or antimicrobial therapies [124]. These infections are now the most common and expensive of all, accounting for 20% of all HAIs and increase the duration of the stay of the patients in the hospital in ICUs [7].

The main factor influencing SSIs is the type of surgery and type of implanted device. According to the ECDC, the most frequently reported types of surgical procedures were hip prosthesis, knee prosthesis, and caesarean section operations. However, the percentage of SSIs in knee prosthesis operation was only 0.5%, but for a laparoscopic colon it reached 10.1% [122].

Alfonso-Sanchez et al. (2017) categorized SSI infections such as environmental contamination or those that depend on the patient’s state of health such as age, sex, transfusion, nutrition, blood albumin levels, etc. [125]. Patients with systemic disease are at higher risk of SSIs, especially those suffering from diabetes mellitus, which weakens antibacterial defense and impairs wound healing. Emergency procedures are also one of the main risk factors for early SSIs compared to elective operations [126,127].

The most frequently reported microorganisms causing SSIs in general are *S. aureus* (21.5%) and *E. coli* (13.9%). The distribution of microorganisms relates to the type of surgical procedure. For open and laparoscopic cholecystectomy and colon operations, the most frequently reported microorganisms were from the family *Enterobacteriaceae*. For all other types of surgical procedure, Gram-positive cocci were more common [122]. According to the Surgical Site Infection Guidelines from the USA, the most common bacteria associated with SSIs are *S. epidermidis*, *S. aureus*, *P. aeruginosa*, and MRSA. The use of antibiotics is accompanied by MDR, manifested mainly in *S. aureus*. These strains form a biofilm which can disperse cells, transferring the infection to a different location in the patient [128]. There are preventive measures such as surgical hand preparation, antibiotic prophylaxis, and postponing surgery in cases of symptomatic infection to avoid SSI [129].

### 3.1. SSI Associated with Orthopedic Implants

Orthopedics deals with bones and joints; millions of people worldwide suffer from joint problems. Bone and joint replacement surgeries are commonly performed on elderly people. Hip prosthesis (HPRO), knee prosthesis (KPRO), and interlocking nails were generally used in orthopedic surgeries. After the surgery, these implants are often problematic and susceptible to many diseases. However, these only affect a minority of patients, with an estimated incidence of 1–9% after primary total arthroplasty [130]. Orthopedic implant infections are categorized into three types: early post-operative infections that usually occur up to 3 months after surgery, delayed infections observed 3–24 months post-surgery, and late post-operative infections occurring more than 24 months post-surgery [131]. In the ECDC epidemiological report, two types of orthopedic surgeries are included: HPRO and KPRO. For HPRO operations, the incidence density of in-hospital SSIs in the periods of 2011–2014 and 2013–2014 was 0.5 and 1.1%, respectively; for KPRO operations, the incidence density of in-hospital SSIs in 2011–2014 and 2013–2014 was 0.2% and 0.6%, respectively [132].

Microorganisms infecting these implants vary according to the anatomical location of the implant in the human body [12]. *Staphylococcus* spp., including MRSA, CoNS, and *E. faecalis*, are the most common infective microorganisms [133]. However, Gram-negative bacteria such as *P. aeruginosa* and *E. coli* cause implant-related infections, but their prevalence is low compared to *S. aureus* strains [134,135,136]. According to pooled data from 13 EU/EU associated countries from a survey during the period of 2013–2014 performed by the ECDC, microorganisms identified in SSIs by surgical procedure type were 65.3% and 69.2% for HPRO and KPRO, respectively. These SSIs were caused by Gram-positive bacteria (mainly *S. aureus* and CoNS, then *Enterococcus* sp. and *Streptococcus* sp.). Of Gram-negative bacteria, the *Enterobacteriaceae* family (*E. coli*, *Enterobacter* sp., *Proteus* sp., *Klebsiella* sp., *Citrobacter* sp., *Serratia* sp.) was the most isolated causal agent of HPRO and KPRO infections, amounting to 18.7% and 15.4%, respectively. Of the other Gram-negative bacteria, *P. aeruginosa* was observed with an incidence of 4.3% in both monitored surgeries (HPRO and KPRO) [132].

The prevalent strategy against orthopedic implant infections includes the usage of antibiotics often accompanied by surgical intervention. Intravenously administered antibiotics, mainly beta-lactams are recommended in higher doses. Subsequently, the oral therapy with antibiotics such as fluoroquinolones, lincosamides, rifampin, tetracycline, linezolid, and metronidazole can be administered. Vancomycin or tobramycin is often used in the powder form [137,138]. Because of various courses of orthopedic infections, there are still some uncertainties, such as way of initial treatment, penetration of antibiotics into the bones, suitability of rifampin or increasing prevalence of MRSA [139].

Many orthopedic infections are associated with biofilms, the curing of which is complicated. Rifampicin is active against biofilms formed by staphylococci/streptococci, while fluoroquinolones are active against biofilms of Gram-negative bacteria. In the case of biofilm, increased doses of drugs as well as a combination of different antibiotics have frequently been administered to patient [140]. The in vitro estimated concentrations of antibiotics inhibiting or killing biofilms have to be 100 to 1000 times higher than the effective MIC for free-floating bacteria [141]. On the other hand, a combination of colistin with fosfomycin demonstrated an inhibitory effect against MDR Gram-negative bacteria forming biofilm [142]. The usual period of treatment of infections associated with biofilm is taken within 6–12 weeks followed by biochemical and radiological monitoring [143].

Various biodegradable carriers have been designed for antibiotics, such as CERAMENT™, which is composed of 40% hydroxyapatite and 60% calcium sulfate. CERAMENT™ is involved in the bone healing process in combination with vancomycin and gentamicin [144]. Gimeno et al. (2018) demonstrated a high effectivity of cefazolin-loaded orthopedic implants [145]. Another important strategy is surface modification using anti-adhesive polymer, nanopatterned surface, hydrogels, biosurfactants, silver ions, and antimicrobial peptides either for the prevention of microbial attachment or the eradication of attached microorganisms [146]. Li et al. (2020) designed alternative strategies to avoid the infection and rejection of titanium implants. They developed titanium implants modified with nitric oxide, and the material demonstrated antimicrobial effectivity by reducing the infection rate of *S. aureus* and *P. aeruginosa* [147].

Johnson et al. (2018) developed a lysostaphin-delivering injectable polyethylene glycol hydrogel. Lysostaphin is an antimicrobial peptide that is predominantly used in soluble form in fracture-related infection, but the introduced technique is unique as it uses a hydrogel for the delivery and encapsulation. Experiments in a murine model have revealed its efficacy by controlling MRSA infection in the fractures compared to the conventional antibiotic treatment. Moreover, a complete healing of the fracture was noticed in 5 weeks, whereas severe infections were observed without the hydrogel carrier [148]. Horprasertkij et al. (2019) used a coating of Schanz pins with known antibiotics in a poly(lactide-co-glycolide) (PLGA) nanosphere spray. The gentamicin and vancomycin PLGA nanospheres were tested in vitro against *S. aureus* and *E. coli*, and the coated pins demonstrated high antimicrobial activity along with excellent biocompatibility [149].

### 3.2. SSI Associated with Cardiovascular Devices

The application of cardiovascular devices (CVDs), such as different types of prosthetic heart valves [150] and cardiac implantable electronic devices (CIEDs), is often the only option for saving a patient’s life [151]. However, people with CVDs are considered at high risk of developing infectious endocarditis (IE), a development of toxic shock associated with high morbidity and a high cost burden [152]. Infection may lead to device removal or lifelong antibiotic treatment.

In 2015, the European Society of Cardiology published guidelines for the management of IEs, which summarized and evaluated all available evidence. There was a similar intent behind the guidelines of the USA dealing with recommendations from the American Heart Association for the prevention of IE [153]. Another document aimed mainly at the antibiotic prophylaxis of IE is the protocol of the National Institute for Health and Clinical Excellence, published in 2008 [154]. The review article of Ivanovic et al. (2019) provides an up-to-date overview of the epidemiology, pathogenesis, and treatment of prosthetic valve endocarditis in European countries [155]. According to the summary of the European Society for Cardiology’s guidelines, the in-hospital mortality rate of patients with IE varies from 15% to 30%. A high mortality rate has been reported in prosthetic valve infections (20–40%), despite these infections only occurring in 1–6% of patients with valve prostheses, with an incidence of 0.3–1.2% per patient year [152]. For the infection of CIEDs, one-year mortality of CIED infection is about 16.9% [156]. Køber et al. (2016) reported an infection rate up to 4.9% over 5 years of follow-up for these type of infections [157].

In general, the risk factors of CVDs depend on the patient’s condition (e.g., age, previous infections and diseases, and use of medication), technical factors (type of device), procedural factors, type of intervention, the infecting organism (mainly *Staphylococcus* sp.), and the echocardiographic findings [158]. In addition, patients undergoing invasive dental procedures are at a higher risk of developing an IE [159].

Microorganisms associated with CIED infections generally attach to a device surface and grow in biofilms. Typical microorganisms are hemolytic streptococci, *Streptococcus gallolyticus* (*Streptococcus bovis*), the HACEK group of bacteria (*Haemophilus* sp., *Aggregatibacter* sp., *Cardiobacterium hominis*, *Eikenella corrodens*, and *Kingella* sp.), *S. aureus*, or community-acquired enterococci [153], but also normal skin flora (e.g., *S. epidermidis*, *Cutibacterium acnes*) [160]. In addition, the development of MDR bacterial strains and drug-tolerant microbial biofilms is not rare [161,162]. Blood culture negative infective endocarditis can be caused by fungi or fastidious bacteria, notably by obligatory intracellular bacteria [163]. Testing for *Coxiella burnetii*, *Bartonella* spp., *Mycoplasma pneumoniae*, *Brucella* spp., and *Legionella pneumophila* should be proposed, followed by atypical bacteria such as *Tropheryma whipplei*, *Bartonella* spp., and fungi (*Candida* spp., *Aspergillus* spp.) from blood cultures [164]. *C. albicans* is the most common yeast associated with vascular CRI. The mortality rate of these infections can reach 50%, even with treatment [165].

As prevention is better than cure, the best way to avoid infections related to CVDs is to reduce the risk by managing the modifiable factors before implantation. These include postponing surgery in cases of fever, the use of external pacing over temporary pacing, avoiding cardiac comorbidity, absolutely sterile operating stations, surgery performed by an experienced surgeon, and the evaluation of antibiotic use [166]. Some therapeutic methods are associated with the maintenance, repair, removal, or replacement of CIEDs. They take advantage of multiple times of washing and cleaning the device, while carriers with a gradual release of antibiotics are applied to the wounds. This technique is preferred in patients who cannot have their CIED replaced, as well as in patients for whom surgery is not possible, or where treatment with conventional antibiotics does not work [167]. Saad and Weiner (2017) noted that it is necessary to avoid the usage of venous hemodialysis catheters in terminal renal disease patients, for who the insertion of a CIED is unavoidable, as it exponentially increases the risk to the patient. In such situations, the use of alternative dialysis methods is to be practiced, resulting in a reduced risk of infectious complications [168]. Another strategy is the usage of drug-eluting antibiotic beads that are inserted directly into the wound, where the antibiotics can perform in the infected location. These beads can be changed every two weeks. Moreover, this technique could be used for patients where other therapies fail, e.g., patients with unremovable implants or in those for which surgery is a risky option [167]. The surface modification of the cardiac implants is another well-known strategy, in which the implants can be coated to provide safe insertion, functioning, and avoiding secondary infections [169,170]. Robotti et al. (2020) used membrane made of cellulose synthesized by *Acetobacter xylinum* as a protection of CIEDs [171]. This highly hydrophilic material was manufactured for biomedical applications as Hylomate^®^. Coupons pre-treated with antibiotics reduced the ability of *S. aureus*, *S. epidermidis*, and *C. acnes* to form biofilms [172].

Tarakji et al. (2019) conducted a clinical trial among patients with CIEDs, in which the patients were divided into two groups: one group received an antibiotic envelope in which a release of minocycline and rifampicin was controlled, while the other group did not receive the antibiotic envelope treatment. This study demonstrated that the use of an antibiotic envelope improved the control of secondary infection among patients compared to the control group. The use of an antibacterial envelope is very promising as it decreased CIED-associated infections by 40% compared to the standard cure [173].

## 4. Conclusions

This review concisely summarizes relevant and up-to-date information on HAIs themselves and HAI-associated microorganisms while also providing a description of several useful approaches for treatment and eradication of HAIs. It is focused on device-associated infections such as CLABSIs including CVCBSIs, CAUTIs, VAPs, and SSIs associated with orthopedic implants and CVDs. Updated information is summarized along with some documents elaborated by the ECDC and CDC. The most relevant microorganisms are mentioned in terms of their frequency of infection associated with medical devices, and they are categorized based on their colonization. These pathogenic microorganisms isolated from the above-mentioned medical devices are summarized in Table 1.

Standard care bundles, conventional therapy, and novel approaches against device-associated infections are briefly mentioned as well. An update of information on HAIs could not only be beneficial for medical professionals but also for researchers to have an idea of the available clinical data and research results, and it could be helpful for improving strategies tackling HAIs.

## Figures and Tables

**Table 1:** Summary of the most frequent HAIs connected to medical devices and associated microorganisms.

Devices-Associated Infections	Microorganisms	References
CVCBSI	CoNS, *S. aureus*, MRSA, *K. pneumoniae*, *P. aeruginosa*, *A. baumannii*, *Candida* spp.	[45,46,47,48,49,54,65]
CAUTI	*E. coli*, enterococci, *P. mirabilis*, *P. aeruginosa*, *K. pneumoniae*, *A. baumannii*, *S. aureus*, *S. epidermidis*, *C. albicans*	[15,29,70,71,76,77]
VAP	*K. pneumoniae*, *S. aureus*, *S. epidermidis*, MRSA, *P. aeruginosa*, *E. coli*, *Candida* spp., *A. fumigatus*	[15,30,97,99,100,102,103,105,106,109]
SSI associated with (a) Orthopedic implants	*S. aureus*, CoNS, MRSA, *Citrobacter* sp. *P. aeruginosa*, *Serratia* sp., *E. coli*, *C. albicans*	[122,128,132,133,134,135,136]
(b) Cardiovascular devices	MRSA, *S. aureus*, CoNS, *P. aeruginosa*, *S. galloliticus*, HACEK group, *C. acnes, Candida* spp., *Aspergillus* spp.	[153,160,161,162,164,165]

## Abbreviations

**Abbreviation**	**Meaning**
BSI	BloodStream Infection
CAUTI	Catheter-Associated Urinary Tract Infection
CDC	Centers for Disease Control and Prevention
CFU	Colony-Forming Unit
CIED	Cardiac Implantable Electronic Device
CLABSI	Central Line-Associated BloodStream Infection
CoNS	Coagulase-Negative Staphylococci
CRBSI	Catheter-Related BloodStream Infection
CRI	Catheter- Related Infection
CVC	Central Venous Catheter
CVCBSI	Central Venous Catheter BloodStream Infection
CVD	CardioVascular Device
ECDC	European Centre for Disease Prevention and Control
EPS	Extracellular Polymeric Substance
EU	European Union
HACEK group	*Haemophilus*, *Aggregatibacter*, *Cardiobacterium*, *Eikenella*, *Kingella* group
HAI	Healthcare-Associated Infection
HAP	Hospital-Acquired Pneumonia
HPRO	Hip PROsthesis
ICU	Intensive Care Unit
IE	Infectious Endocarditidis
KPRO	Knee PROsthesis
MDR	Multi-Drug Resistant/Resistance
MIC MRSA	Minimal Inhibitory Concentration Methicillin-Resistant *Staphylococcus aureus*
SNACET	S-nitroso-N-acetyl-l-cysteine ethyl ester
SSI	Surgical Site Infection
UTI	Urinary Tract Infection
VAP	Ventilator-Associated Pneumonia
WHO	World Health Organization
